# Supplementary material for: Biocompatibility and Biological Effects of Surface-Modified Conjugated Polymer Nanoparticles
Source: Molecules. 2023 Feb 21;28(5):2034. doi: 10.3390/molecules28052034 (PMC10003845; doi:10.3390/molecules28052034)
Supplement: Supplementary file 1 [file molecules-28-02034-s001.zip › molecules-2215177-supplementary.pdf]

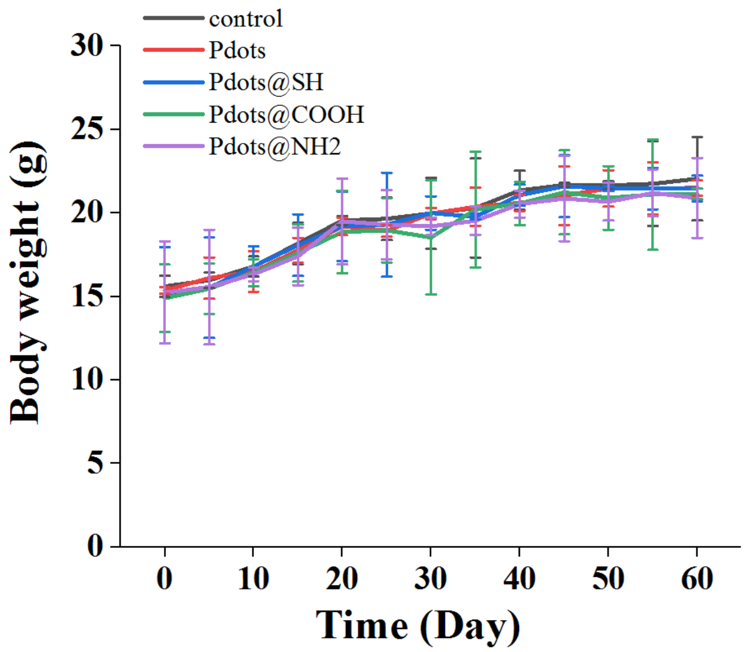

Supplementary Figure S1. Body weight change curve of mice injected with Pdots, Pdots@SH, Pdots@COOH and Pdots@NH_2 by tail vein at different time points.

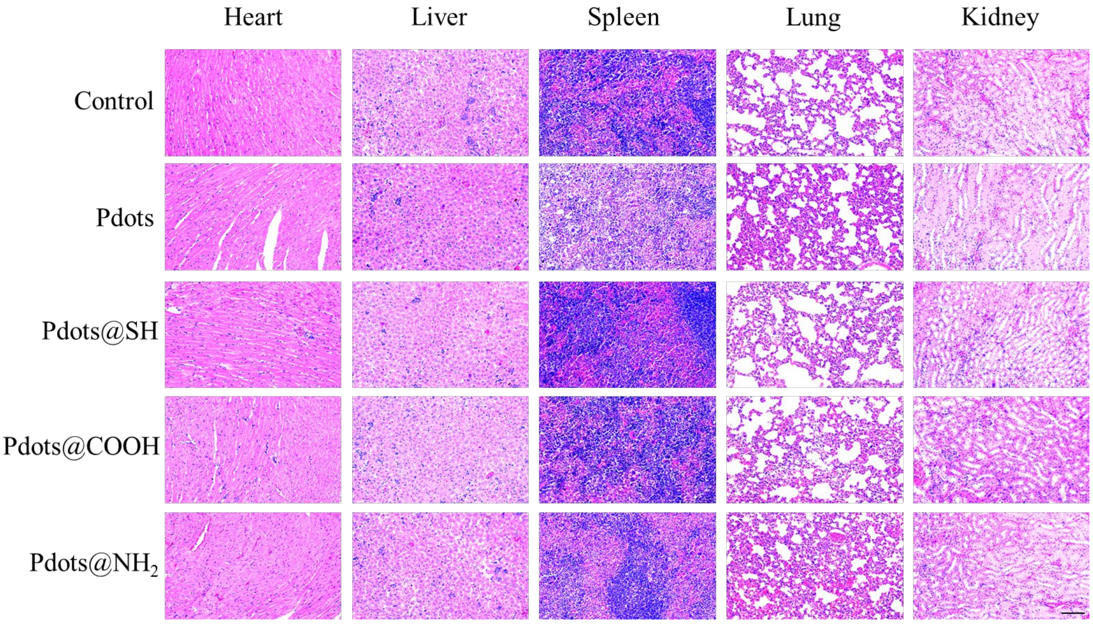

Supplementary Figure S2. Hematoxylin and eosin-stained images of major organs including the heart, liver, spleen, lungs and kidneys from the Balb/c mice injected with Pdots, Pdots@SH, Pdots@COOH and Pdots@NH$_2$ at 7 days.

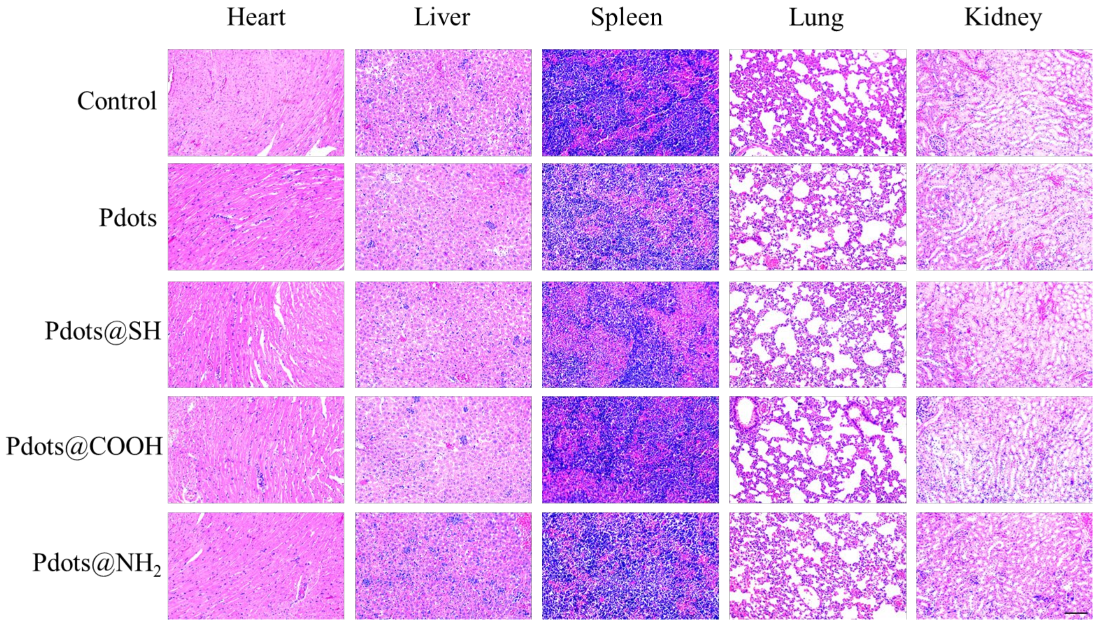

Supplementary Figure S3. Hematoxylin and eosin-stained images of major organs including the heart, liver, spleen, lungs and kidneys from the Balb/c mice injected with Pdots, Pdots@SH, Pdots@COOH and Pdots@NH_2 at 15 days.
